# Neuron-Derived Estrogen Regulates Synaptic Plasticity and Memory

**DOI:** 10.1523/JNEUROSCI.1970-18.2019

**Published:** 2019-04-10

**Authors:** Yujiao Lu, Gangadhara R. Sareddy, Jing Wang, Ruimin Wang, Yong Li, Yan Dong, Quanguang Zhang, Jinyou Liu, Jason C. O'Connor, Jianhua Xu, Ratna K. Vadlamudi, Darrell W. Brann

**Affiliations:** ^1^Department of Neuroscience and Regenerative Medicine, Medical College of Georgia, Augusta University, Augusta, Georgia 30912,; ^2^Department of Obstetrics and Gynecology, University of Texas Health, San Antonio, Texas 78229,; ^3^North China University of Science and Technology, Neurobiology Institute, Tangshan, PR China,; ^4^Department of Pharmacology, School of Medicine, UT Health San Antonio, San Antonio, Texas 78229, and; ^5^Audie L. Murphy VA Hospital, South Texas Veterans Health System, San Antonio, Texas 78229

**Keywords:** aromatase, cerebral cortex, cognition, estrogen, hippocampus, synapse

## Abstract

17β-estradiol (E2) is produced from androgens via the action of the enzyme aromatase. E2 is known to be made in neurons in the brain, but its precise functions in the brain are unclear. Here, we used a forebrain-neuron-specific aromatase knock-out (FBN-ARO-KO) mouse model to deplete neuron-derived E2 in the forebrain of mice and thereby elucidate its functions. FBN-ARO-KO mice showed a 70–80% decrease in aromatase and forebrain E2 levels compared with FLOX controls. Male and female FBN-ARO-KO mice exhibited significant deficits in forebrain spine and synaptic density, as well as hippocampal-dependent spatial reference memory, recognition memory, and contextual fear memory, but had normal locomotor function and anxiety levels. Reinstating forebrain E2 levels via exogenous *in vivo* E2 administration was able to rescue both the molecular and behavioral defects in FBN-ARO-KO mice. Furthermore, *in vitro* studies using FBN-ARO-KO hippocampal slices revealed that, whereas induction of long-term potentiation (LTP) was normal, the amplitude was significantly decreased. Intriguingly, the LTP defect could be fully rescued by acute E2 treatment *in vitro*. Mechanistic studies revealed that FBN-ARO-KO mice had compromised rapid kinase (AKT, ERK) and CREB-BDNF signaling in the hippocampus and cerebral cortex. In addition, acute E2 rescue of LTP in hippocampal FBN-ARO-KO slices could be blocked by administration of a MEK/ERK inhibitor, further suggesting a key role for rapid ERK signaling in neuronal E2 effects. In conclusion, the findings provide evidence of a critical role for neuron-derived E2 in regulating synaptic plasticity and cognitive function in the male and female brain.

**SIGNIFICANCE STATEMENT** The steroid hormone 17β-estradiol (E2) is well known to be produced in the ovaries in females. Intriguingly, forebrain neurons also express aromatase, the E2 biosynthetic enzyme, but the precise functions of neuron-derived E2 is unclear. Using a novel forebrain-neuron-specific aromatase knock-out mouse model to deplete neuron-derived E2, the current study provides direct genetic evidence of a critical role for neuron-derived E2 in the regulation of rapid AKT-ERK and CREB-BDNF signaling in the mouse forebrain and demonstrates that neuron-derived E2 is essential for normal expression of LTP, synaptic plasticity, and cognitive function in both the male and female brain. These findings suggest that neuron-derived E2 functions as a novel neuromodulator in the forebrain to control synaptic plasticity and cognitive function.

## Introduction

It is well established that the brain is a target organ of the steroid hormone 17β-estradiol (E2), which is traditionally believed to be generated principally by the ovaries in females. E2 has been implicated to exert both neurotrophic and neuroprotective actions in the brain, including modulation of reproductive behavior, synaptic plasticity, neuroprotection, and cognition (Li et al., 2004; Brann et al., 2007; Simpkins et al., 2012; Khan et al., 2013). Until recently, the commonly accepted belief in the field has been that gonadal-derived E2 is responsible for the above-described actions in the brain. However, studies in birds, rodents, monkeys, and humans have found that E2 is also produced in the male and female brain, with high concentrations produced in the hypothalamus, amygdala, hippocampus, and cerebral cortex due to expression of the biosynthetic enzyme aromatase, which converts testosterone to E2 (Stoffel-Wagner et al., 1999; Hojo et al., 2004). A number of studies have reported that aromatase is significantly expressed in neurons in the above listed brain regions, with subcellular localization noted in the cell soma and neurites, especially at presynaptic terminals (Saldanha et al., 2011). Additional work has shown that either no or low expression of aromatase is detected in astrocytes basally (Yague et al., 2008; Zhang et al., 2014); however, aromatase expression in astrocytes can be significantly induced after brain injury or various stressors (Peterson et al., 2001; Zhang et al., 2014). Intriguingly, aromatase inhibitor treatment in either cultured mouse hippocampal neurons or in ovariectomized (ovx) mice *in vivo* has been reported to result in a significant decrease in dendritic spine formation and synapse loss (Kretz et al., 2004; Rune and Frotscher, 2005). Aromatase inhibitor administration *in vivo* has also been reported to induce memory defects in humans, mice, and songbirds (Oberlander et al., 2004; Tuscher et al., 2016; Bender et al., 2007; Phillips et al., 2011; Underwood et al., 2018).

Collectively, these localization and pharmacological inhibitor studies have raised the possibility that neuron-derived E2 may function as a neuromodulator in the brain. However, a limitation of the pharmacological inhibitor studies is that the inhibitors may have “off-target” actions that could alternatively explain their effects. Furthermore, aromatase inhibitors are not cell specific and their administration *in vivo* can inhibit both neuronal and astrocyte-derived E2 production, making it difficult to differentiate the role of neuronal- versus astrocyte-derived E2 in certain situations. To address these potential limitations and to more definitively address the role of neuron-derived E2 in the brain, we generated novel forebrain-neuron-specific aromatase knock-out (FBN-ARO-KO) mice to deplete neuron-derived E2 in the forebrain. Using these mice, we show that neuron-derived E2 is critical for normal activation of rapid AKT-ERK kinase signaling and CREB-BDNF neurotrophic signaling in the forebrain. Our study further demonstrates that neuron-derived E2 is essential for normal LTP and that this effect is dependent upon rapid ERK signaling. In addition, the findings of our study revealed that neuronal E2 has an important role in the regulation of spine density, synapse formation, and hippocampal-dependent cognitive function in both the male and female forebrain.

## Materials and Methods

### 

#### 

##### Generation of the FBN-ARO-KO mouse model.

To determine the role of forebrain-neuron-derived E2 in the modulation of synaptic plasticity and cognitive behavior, we custom created a FBN-ARO-KO mouse using Genoway (Lyon, France). Transgenic mice that expressed a floxed allele of *Cyp19a1* (aromatase) were generated using a site-specific Cre/loxP and FLP/FRT recombination system to facilitate the conditional aromatase KO *in vivo*. The Cre-LoxP recombination system has been widely used to selectively knock out genes *in vivo*. To specifically knock out the *Cyp19a1* gene in forebrain neurons, we targeted the *Cyp19a1* gene by inserting two loxP sites flanking *Cyp19a1* exons 10 and 11. Removal of *Cyp19a1* exons 10 and 11 results in conditional deletion of 3318 bp of the coding sequence encoding for the second substrate-binding site, the iron-binding site, and part of the p450 domain and the poly A signal. The targeting construct was transfected into 129/sv ES cells, which were then injected into C57BL/6J blastocysts using standard protocols. Breeding of chimera male with C57BL6 FLP-deleter mice was performed to generate heterozygous mice (mosaic) carrying the Neo-excised conditional KO allele (floxed mice). To conditionally delete locally synthesized aromatase in forebrain neurons, we next generated *Cyp19a1 Cre/LoxP* conditional KO mice, under the control of the Ca^2+^-/calmodulin-dependent protein kinase II α (*CaMKII*α) promoter, which expresses *Cre* exclusively in forebrain excitatory neurons. *CaMKII*α-*Cre* mice were purchased from The Jackson Laboratory. FLOX and FBN-ARO-KO animals were maintained in the C57BL/6 background. Because of the potential for recombination in Sertoli cells of *CaMKII*α-*Cre*^+^ mice, needed mice were generated by breeding *CaMKII*α-*Cre*^+^ females with *CaMKII*α-*Cre*^−^ males. Mice were genotyped by tail digestion, followed by genomic DNA PCR using following primers. FLOX-forward: 5′-GCCATATTCCTGCAACAGTTTATTTGAGG-3′; FLOX-reverse: 5′-GTAAAACCATTCCTGGAAAATTCATAACAACC-3′; Cre-forward: 5′-GCGGTCTGGCAGTAAAAACTATC-3′; Cre-reverse: 5′-GTGAAACAG CATTGCTGTCACTT-3′; Cre-mediated excision of *Cyp19a1* exons 10 and 11 resulted in generation of a unique 775 bp band that was determined using the following primers: Cre excised FLOX-forward: 5′-GCATGATCTCATCGACTCTGTAGCAGC-3′; Cre excised FLOX-reverse: 5′-ATGAGCAAGTGTGCCTATGCCAAGC-3′. Animal experiments and handling were done in accordance with ethical guidelines of Declaration of Helsinki and the National Institutes of Health and were approved by the Augusta University and UT Health San Antonio Institutional Animal Care and Use Committees.

##### Tissue lysate preparation and Western blotting.

Three- to 5-month-old male and female FLOX and FBN-ARO-KO mice were used in the study. Mice were killed under deep anesthesia at different time points, as detailed in the experiment descriptions. Mice were perfused with ice-cold 0.9% saline and the whole brain was quickly removed, with cortex, hippocampus, and cerebellum isolated and separately stored at −80°C until use. For lysate preparation, tissues were homogenized as we described previously (Lu et al., 2017a). Briefly, homogenization was performed using a motor-driven Teflon homogenizer in 400 μl of ice-cold homogenization buffer (Thermo Scientific) containing protease and phosphatase inhibitors according to the manufacturer's protocol. The homogenates were vigorously mixed for 20 min on a rotator at 4°C and centrifuged at 12,000 × *g* for 30 min to get total protein fractions in the supernatant. Total protein concentration was determined using Protein Assay Dye Reagent Concentrate (Bio-Rad). Samples were stored at −80°C until use. Western blotting was performed as we described previously (Sareddy et al., 2015). Primary antibodies used for Western blotting analysis include: anti-PSD95 (Transduction Laboratories, catalog #P43520, 1:1000), anti-synaptophysin (Cell Signaling Technology, catalog #4329S, 1:1000), anti-p-AKT (Cell Signaling Technology, catalog #4060S, 1:1000), anti-AKT (Cell Signaling Technology, catalog #4691P, 1:1000), anti-p-ERK1/2 (Cell Signaling Technology, catalog #9101S, 1:1000), anti-p-CREB (Cell Signaling Technology, catalog #9198S, 1:1000), anti-ERK1/2 (Biosource, catalog #0703, 1:1500), anti-CREB (Santa Cruz Biotechnology, catalog #A2715, 1:200), anti-GAPDH (Santa Cruz Biotechnology, catalog #H2114, 1:2000), anti-BDNF (EMD Millipore, catalog #2865371, 1:1000), anti-aromatase (Invitrogen, catalog #SD2376082A, 1:1000), and anti-β-actin (Sigma-Aldrich, catalog #014M4759, 1:5000). Bound proteins were visualized using a CCD digital imaging system and the band intensities were quantified using ImageJ analysis software version 1.49. Band densities for the indicated proteins were normalized to the corresponding loading controls.

##### Brain section preparation and immunofluorescence staining.

Mouse brain sections were prepared as previously described (Lu et al., 2017b). Briefly, mice were transcardially perfused with ice-cold 0.9% saline under deep anesthesia. The mice were then killed by decapitation with the whole brain quickly removed and postfixed in 4% paraformaldehyde at 4°C for 48 h, and cryoprotected with 30% sucrose. Frozen-sections of 30 μm thickness were cut in a series on a Leica Rm2155 microtome. The collected sections were saved in stock solution for subsequent staining. For immunofluorescence staining, brain floating sections were incubated with 10% normal donkey serum for 1 h at room temperature, followed by incubation with appropriate primary antibodies overnight. The primary antibodies used in this study included: anti-aromatase (Invitrogen, catalog #SD2376082A, 1:200), anti-estrogen receptor-β (anti-ER-β) (Invitrogen, catalog #RH238843, 1:200), anti-E2 (Abcam, catalog #131413, 1:100), anti-GFAP (Abcam, catalog #53554, 1:500), anti-synaptophysin (Cell Signaling Technology, catalog #4329S, 1:200), anti-p-AKT (Cell Signaling Technology, catalog #4060S, 1:200), anti-p-ERK/1/2 (Cell Signaling Technology, catalog #9101S, 1:200), anti-p-CREB (Cell Signaling Technology, catalog #9198S, 1:150), anti-PSD95 (Transduction Laboratories, catalog #P43520, 1:200), anti-BDNF (EMD Millipore, catalog #2865371, 1:200), anti-NeuN (EMD Millipore, catalog #mAB377, 1:400), anti-ER-α (EMD Millipore, catalog #2775528, 1:300). Sections were subsequently washed for three times with 0.1% Triton X-100 solution at room temperature, followed by incubation with proper Alexa Fluor 568/488 donkey anti-mouse/rabbit/goat secondary antibody (Thermo Fisher) for 1 h. After another three washes, the sections were mounted and coverslipped in Vectashield mounting medium with DAPI (Vector Laboratories). Fluorescent images were captured on an LSM510 meta confocal laser microscope (Carl Zeiss) using 40–100× oil-immersion Neofluor objectives with the image size set at 1024 × 1024 pixels. The captured images were analyzed using LSM510 Meta imaging software. At least four to five representative sections were used per animal for immunostaining and the typical staining was selected for presentation.

##### Measurement of E2 levels.

17β-Estradiol levels in hippocampal CA1, cortex, and serum were measured using a high-sensitivity ELISA kit (ADI-900-174, Enzo Life Sciences), as we described previously (Zhang et al., 2014). Briefly, 100 μl of sample was added into the bottom of an appropriate well coated with a donkey anti-sheep polyclonal antibody. Subsequently, 50 μl of E2 conjugate was added, followed by 50 μl of sheep polyclonal antibody, to E2. The plate was then sealed and incubated at room temperature with shaking speed of ∼500 rpm for 2 h. After washing 3 times with 400 μl of wash buffer each time, 200 μl of pNpp substrate was added into each well and incubated for 1 h at room temperature without shaking. Afterward, 50 μl of stop solution was added into each well and the optical density was read at 405 nm. 17β-Estradiol level in each sample was calculated according to the established standard curve and expressed as percentage change versus FLOX mice.

##### Barnes maze task.

The Barnes maze task was used in the current study to examine hippocampal-dependent spatial learning and memory (Lu et al., 2017a). Briefly, this behavior task was divided into two stages: a training trial and a probe trial. On each day of the training trial stage (days 1–3), an overhead video camera controlled by ANY-maze video-tracking software (Stoelting) was used to record the escape latency of mice from the center of platform to the hidden chamber, their escape velocity, as well as their escape traces. The probe test was performed on day 4 with the hidden chamber removed. During the 90 s time period, the time spent in the target quadrant where the hidden box had been and the searching errors were recorded. Searching error was defined as exploring any hole that did not have a target chamber under it. The escape latency, escape velocity, quadrant occupancy, and searching errors were quantified afterward using ANY-maze software. In the E2 replacement experiment, only the escape latency and quadrant occupancy in the probe trial were selected for analysis.

##### Novel object recognition test.

The novel object recognition test was used to examine the alteration of recognition memory, a hippocampal-dependent working memory (Lu et al., 2017a). One day before the test, mice were allowed 5 min to explore the empty arena for habituation. The test was subsequently divided into two stages. In stage 1, the mice were placed in the same recognition box containing two identical objects at an equal distance for 5 min exploration. In stage 2, 24 h later, the mice were returned to the recognition box in the presence of one familiar object and one novel object to test the long-term recognition memory. The novel object had the same height and volume, but different shape and appearance as the familiar object. Exploration of the object was defined as the animal's nose being within a zone 2 cm from the object. Time spent exploring each object, recorded using ANY-maze video-tracking software as mentioned above, and the discrimination index (the percentage of time spent exploring the novel object) were analyzed afterward.

##### Fear-conditioning test.

Fear conditioning was performed as described previously (Li and Kim, 2017). Briefly, a fear-conditioning chamber (17*17.5*33 cm^3^) was used for conditioning and testing. The walls of the chamber were made of black laminated particle boards and the floor was made of a grid of steel rods (diameter, 3.2 mm) for electric shocks. The chamber was illuminated with 36 lux white light. A camera was mounted above the chamber for recording. The task was divided into two sessions: conditioning test (day 1) and fear memory test (day 2). At day 1, mice were placed in the conditioning chamber for a 2 min acclimation, followed by three 30 s tones (2.8 kHz sine waves and presented at 90 dB) co-terminated with a 2 s shock at 0.5 mA for every 2 min. Mice were returned to their home cages immediately after conditioning. The conditioning chamber was cleaned with 70% ethanol after each animal testing. The fear acquisition curve at this stage was analyzed according to the freezing rate during each tone presentation. At day 2, contextual fear memory was evaluated by putting the mice into the same conditioning chamber for 5 min without shock. Freezing time was recorded during this period. Cued fear memory was tested 1 h later with the visual context changed by lining the walls and floor of the chamber with paper. Mice were put in the chamber for 90 s, followed by three 30 s tones every 2 min. Freezing time during the tones was recorded. Freezing behavior is counted as unnoticeable movement for ≥ 1 s excluding the respiration movement.

##### Forced-swimming test.

The forced-swimming test was used to measure depressive-like behavior (Xu et al., 2017). Mice were put in a 2 L beaker filled with water of 25 ± 1°C to 4.5 cm from the top, which ensured that the mice could not touch the bottom with their tails or escape from the top. When mice were put into the beaker, the recording time was started and the duration was set to 6 min. Immobility time during each test was recorded as lack of movement (except that required to keep the mouse afloat and breathing). After the test, mice were gently warmed under a heating lamp and returned to their home cages.

##### Open-field test.

The open-field test was used to examine locomotor function and anxiety, as described previously with minor revisions (Christoffel et al., 2012). A black open box with 56 cm*56 cm was used. To start the test, a mouse was placed at one corner of the box, making it face the wall. During a 5 min period, the numbers of episodes of rearing and grooming, the travel distance, and the time spent in a delineated center zone (26*26 cm) were recorded.

##### Spine density analysis.

To look for changes in neuronal morphology and spine density in FLOX and FBN-ARO-KO mice, the FD Rapid GolgiStain kit (FD NeuroTechnologies) was used according to the manufacturer's instructions. Briefly, mice were killed under deep anesthesia. The brains were quickly collected without perfusion and rinsed in double-distilled water to remove blood from the surface. Brain tissues were subsequently immersed in the impregnation solution made by mixing equal volumes of solutions A and B and stored at room temperature for 10 d in the dark. Subsequently, the brains were transferred into solution C and stored at 4°C in the dark for 4 d. Sections of 200 μm thickness were cut on a vibratome and mounted on gelatin-coated slides with solution C for natural drying at room temperature for 2 d. Dried sections were further processed according to the manufacturer's instructions. Images of dendrites in the hippocampus and cortex (primary somatosensory cortex) were captured on an Olympus IX70 microscope using a 100× objective. Spines along the apical dendrite were selected for analysis. Spines were classified as mushroom, thin and stubby, which were defined as follows. If the “neck ratio” (head diameter/neck diameter) of a spine was >1.1, then the spine was classified as mushroom or thin; it was mushroom when the head diameter was ≥0.3 μm and thin otherwise. If the neck ratio of a spine was ≤1.1, then the spine was classified as thin or stubby; if the “thin ratio” (spine length/head diameter) was >2.5, then it was classified as thin and stubby otherwise (Kim and Li, 2015). Spine density was analyzed as spine numbers per micrometer. For one brain section, spine densities of dendrites from five separate neurons in both hippocampus and cortex were measured, with the mean value presented as a single value for one animal. Spine density was analyzed using ImageJ software.

##### Synapse density analysis.

ImageJ software was used for the measurement of synaptophysin and PSD95 intensities to determine the numbers of presynaptic and postsynaptic puncta, respectively. To accurately assess SYN/PSD95 contact, synaptophysin and PSD95 paired puncta were manually counted under a 100× objective of LSM510 Meta confocal laser microscope (Carl Zeiss) (Cao et al., 2013). Synaptic density in hippocampal CA1 and cortex regions was quantified as puncta per cubic micrometer and presented as percentage changes versus FLOX control.

##### Electrophysiology.

Acute dorsal hippocampal slices of 400 μm prepared from 2- to 3-month-old FBN-ARO-KO mice and their FLOX littermates were perfused with ACSF at 32 ± 0.5°C in a recording chamber at a rate of 1.6 ml/min (Li and Kim, 2016). Field EPSPs (fEPSPs) from the hippocampal CA1 region were recorded when stimulating the Schaffer collateral axons. For the examination of I/O relationships, fEPSPs were recorded with a set of four presynaptic stimulus intensities, which were then selected for linear I/O relationships. For baseline fEPSP recording, the stimulation strength was adjusted to 50% of the maximal fEPSP amplitude. After a 20 min baseline recording, LTP was induced with high-frequency stimulation (HFS; 100 Hz, 1 s, 20 s intervals) and recorded for 60 min. The mean fEPSP slope between 50 and 60 min after LTP induction was selected for quantitative analysis. fEPSP slope from FBN-ARO-KO group was normalized to FLOX control and expressed as percentage change compared with the FLOX control group. For the *in vitro* E2 rescue experiment, E2 was dissolved in DMSO, diluted to working concentration (1 nm) in oxygenated ACSF (Di Mauro et al., 2015). A DMSO (0.001%) vehicle control was also included in the experiment. In addition, the MEK inhibitor, U0126 (Cell Signaling Technology, catalog #9903S, 10 μm) was also dissolved in DMSO and diluted to working concentration in oxygenated ACSF. U0126 was coadministered with E2. The drugs were applied for all the recording period 20 min before the application of the stimulation protocol.

##### *In vivo* E2 rescue experiment.

Three-month-old ovx female mice were used in this experiment, which were divided into four groups: FLOX + placebo, FLOX + E2, FBN-ARO-KO + placebo, and FBN-ARO-KO + E2. Alzet minipumps osmotic minipumps; model 1007D, 7 d release; Durect) with placebo or E2 (0.0167 mg) were implanted subcutaneously in the upper midback region at the time of ovariectomy. The dose of E2 used here yields stable serum levels of 26.52 ± 0.89 pg/ml, which represents a physiological diestrus II–proestrus level of E2 (Nelson et al., 1981). Molecular and functional endpoints were examined 7 d after minipump implantation.

##### Experimental design and statistical analyses.

All quantitative analyses were performed on age-matched FLOX control and FBN-ARO-KO mice. Except for the *in vivo* E2 rescue experiment, both male and female mice were used. Only female mice were used for *in vivo* E2 replacement. For behavioral tests, 8–10 mice were used for each group; otherwise, 4–6 samples from each group were analyzed. SigmaStat 3.5 software was used to analyze all data. Data represented in bar graphs were expressed as mean ± SE. A Student's *t* test was performed when only comparing two groups. Statistical data from the Barnes maze training trial, fear acquisition test, electrophysiological measurements, and part of the *in vivo* E2 replacement experiments requiring multiple groups comparisons were analyzed with two-way ANOVA followed by Tukey's all pairwise comparisons test to determine group differences. A value of *p* < 0.05 was considered statistically significant.

## Results

### Characterization of FBN-ARO-KO Mice

**Figure 1.**
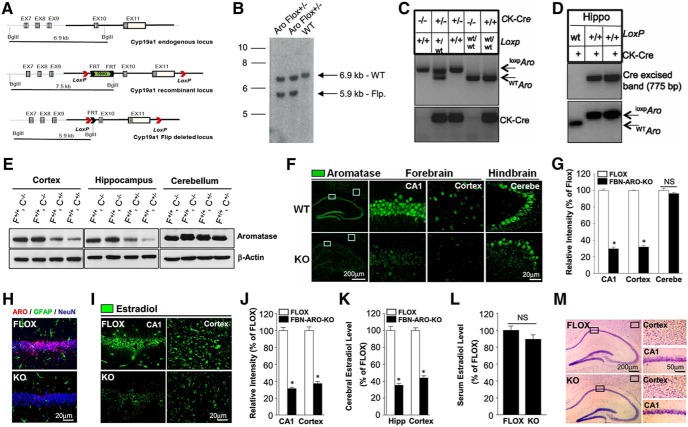
Generation and characterization of male FBN-ARO-KO mice. ***A***, Schematic representation of endogenous (WT) and recombinant locus of mouse *CYP19a1*. ***B***, Confirmation of the excision of Neo cassette by Southern blotting of genomic DNA. ***C***, Tail DNA PCR confirmation of bitransgenic FBN-ARO-KO mice expressing CaMKIIα-Cre and ^loxp^*CYP19a1*. ***D***, Confirmation of excision of floxed region in hippocampus of FLOX control and FBN-ARO-KO mice using genomic PCR. ***E***, Total lysates of cortex, hippocampus, and cerebellum collected from FLOX control and homozygous FBN-ARO-KO (*CYP19a1* loxp/loxp, CaMKIIα-Cre^+/−^) male mice were subjected to Western blotting with Aromatase antibody. F, FLOX; C, Cre. ***F***, IHC confirmation of aromatase expression in hippocampal CA1 region, cerebral cortex, and cerebellum of FLOX control and FBN-ARO-KO mice. ***G***, Relative intensity of aromatase in FBN-ARO-KO mice compared with FLOX control was quantified following confocal analysis above. ***H***, Confirmation of neuron-specific expression of aromatase in the forebrain by aromatase, GFAP and NeuN costaining. ***I***, ***J***, Examination of E2 levels in hippocampal CA1 subregion and cortex by IHC (***I***), results that were further quantified as percentage changes versus Flox mice (***J***). ***K***, E2 levels in the forebrain were confirmed by E2 ELISA assay. ***L***, Serum E2 levels were furthered assayed by E2 ELISA to determine the potential impact of neuroestradiol loss on E2 circulating levels. ***M***, Nissl staining of coronal sections from both FLOX and FBN-ARO-KO mice was conducted to examine the potential change of brain structure in FBN-ARO-KO mice. Bar graphs represent mean ± SEM. *n* = 4–5. *p < 0.05.

A transgenic mouse that expresses a floxed allele of *Cyp19a1* (aromatase) was initially established to facilitate conditional knock out of aromatase *in vivo* using a site-specific Cre/loxP recombination system. We targeted the *Cyp19a1* gene by inserting two loxP sites flanking *Cyp19a1* exons 10 and 11 (Fig. 1*A*). Removal of *Cyp19a1* exons 10 and 11 results in conditional deletion of 3318 bp of the coding sequence encoding for the second substrate-binding site, the iron-binding site, part of the p450 domain, and the poly A signal. Breeding of chimera males with C57BL6 FLP-deleter mice was used to generate Neo-excised conditional KO allele (floxed mice). Excision of the Neo cassette was confirmed by Southern blotting of genomic DNA (Fig. 1*B*). *CaMKII*α-*Cre*^−^ males were bred with *CaMKII*α-*Cre*^+^ females that expressed *Cre* under the control of the *CaMKII*α promoter, which is specially expressed in excitatory neurons of the forebrain by postnatal days 18–23 (Tsien et al., 1996), thus allowing selective KO of aromatase in forebrain excitatory neurons. Mice were genotyped with PCR on genomic DNA of tail biopsies to confirm the genotype of floxed and Cre alleles (Fig. 1*C*). FBN-ARO-KO mice are viable and fertile, with no obvious gross immunological, reproductive, or neurological abnormality or phenotype.

**Figure 2.**
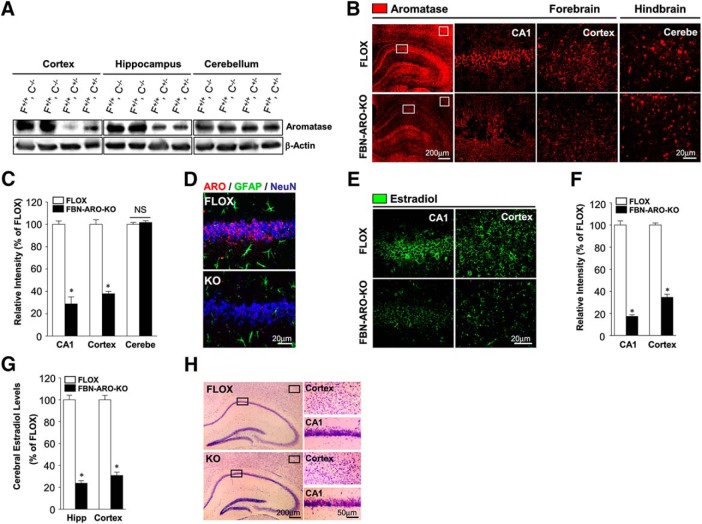
Characterization of ovx female FBN-ARO-KO mice. ***A***, Western blot was conducted to examine aromatase expression in hippocampus, cortex, and cerebellum from both ovx female FLOX and FBN-ARO-KO mice. ***B***,***C***, Confirmation of aromatase levels in forebrain and hindbrain by IHC (***B***), a result that was further quantitatively analyzed (***C***). ***D***, Neuronal-specific localization of aromatase was confirmed by costaining for aromatase, GFAP, and NeuN. ***E***, ***F***, IHC analysis (***E***) and further quantification (***F***) of E2 levels in hippocampal CA1 region and cortex. ***G***, ELISA assay was performed to confirm significant decrease of forebrain E2 levels in ovx female FBN-ARO-KO mice. ***H***, Nissl staining of coronal sections confirming normal brain structure following forebrain neuronal E2 depletion. Data are shown as means ± SEM of determinations from each group. *n* = 5. *p < 0.05 versus FLOX group. Cerebe, Cerebellum.

We next confirmed the KO effectiveness in the *CaMKII*α-*Cre*^+/−^ mice. As shown in Figure 1*D*, PCR showed that deletion of *Cyp19a1* exons 10 and 11 resulted in generation of a unique 775 bp band due to expression of *CaMKII*α-*Cre* in the forebrain and excision of the floxed region. However, *CaMKII*α-*Cre* expression did not affect *Cyp19a1* gene size in WT mice, so no 775 bp product can be seen (Fig. 1*D*, lane 1, top). In addition, Western blot analysis revealed a robust decrease of aromatase expression in the forebrain (hippocampus and cortex), but not in the hindbrain (cerebellum) of both male FBN-ARO-KO mice (Fig. 1*E*) and ovx female FBN-ARO-KO mice (Fig. 2*A*) compared with the FLOX controls. Likewise, immunohistochemistry (IHC) for aromatase showed a 65–70% decrease in the hippocampal CA1 region and cortex of both male FBN-ARO-KO mice (Fig. 1*F*,*G*, *p* < 0.001, unpaired *t* test) and ovx female FBN-ARO-KO mice (Fig. 2*B*,*C*, *p* < 0.001, unpaired *t* test) compared with FLOX mice, with no decrease observed in the hindbrain (*p* = 0.2346 and *p* = 0.5022, respectively, unpaired *t* test).

We next performed triple IHC with antibodies to aromatase (red), the astrocyte marker GFAP (green), and the neuronal marker NeuN (blue), which revealed that in both male FLOX mice (Fig. 1*H*) and ovx female FLOX mice (Fig. 2*D*), aromatase is specifically expressed in neurons in the hippocampal CA1 region, but not in astrocytes, and that FBN-ARO-KO mice display a profound loss of aromatase expression in hippocampal CA1 neurons, verifying the neuron-specific KO of aromatase. The small residual aromatase in the FBN-ARO-KO mouse may be due to aromatase expression in inhibitory neurons and interneurons or a small percentage of excitatory neurons that lack *Cre* expression.

Next, we determined E2 levels in the forebrain in male FBN-ARO-KO mice using IHC (Fig. 1*I*,*J*) and ELISA (Fig. 1*K*). Consistent with the KO results for aromatase expression, IHC and ELISA revealed that neuron-derived E2 was decreased by 65–70% in the hippocampal CA1 region and cortex of male FBN-ARO-KO mice (Fig. 1*I–K*, *p* < 0.001, unpaired *t* test) and in ovx female FBN-ARO-KO mice (Fig. 2*E–G*, *p* < 0.001, unpaired *t* test) compared with FLOX mice. In contrast, there was no significant difference in serum E2 levels between FLOX and FBN-ARO-KO mice (Fig. 1*L*, *p* = 0.1838, unpaired *t* test), demonstrating that the KO was specific for forebrain E2 levels and did not affect circulating E2 levels. Finally, Nissl staining revealed that postnatal deletion of the aromatase gene in forebrain excitatory neurons did not result in any apparent defects in brain structure in male FBN-ARO-KO mice (Fig. 1*M*) or ovx female FBN-ARO-KO mice (Fig. 2*H*).

### Cognitive function is impaired in FBN-ARO-KO mice

**Figure 3.**
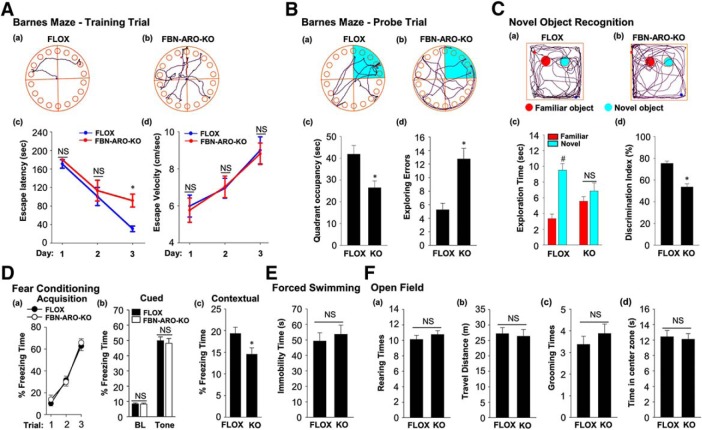
Male FBN-ARO-KO mice exhibit cognitive defects but normal locomotor activity and anxiety levels. ***A***, Representative tracking plots of FLOX (***Aa***) and FBN-ARO-KO (***Ab***) mice on the third day of the Barnes maze training trial. Escape latency (***Ac***) and escape velocity (***Ad***) were also recorded and statistical data are shown. ***B***, Representative tracking plots of FLOX (***Ba***) and FBN-ARO-KO (***Bb***) mice on the probe trial day. Quadrant occupancy (***Bc***) and exploring errors (***Bd***) made during probe trial stage were recorded and statistically analyzed. ***C***, The novel object recognition test (NORT) was performed to evaluate recognition memory. Representative tracking plots of the indicated mice on NORT choice day are shown in ***Ca*** and ***Cb***. Exploration time (***Cc***) and discrimination index (***Cd***) were statistically analyzed afterward. ***D***, Fear acquisition curve during conditioning test was analyzed (***Da***). Cued freezing rate (***Db***) and contextual freezing rate (***Dc***) were assayed 24 h after fear conditioning to test cued fear memory and contextual fear memory, respectively. ***E***, The forced-swimming test was conducted to evaluate the depression level. Immobility time during forced swimming was recorded as an index of depression. ***F***, Rearing times (***Fa***), travel distance (***Fb***), grooming times (***Fc***), and time in center zone (***Fd***) were determined in the open-field test to assess locomotor function (***Fa***, ***Fb***) and anxiety level (***Fc***, ***Fd***). Data are shown as means ± SEM. *n* = 8. *p < 0.05 versus FLOX group; #*p* < 0.05 versus familiar object.

**Figure 4.**
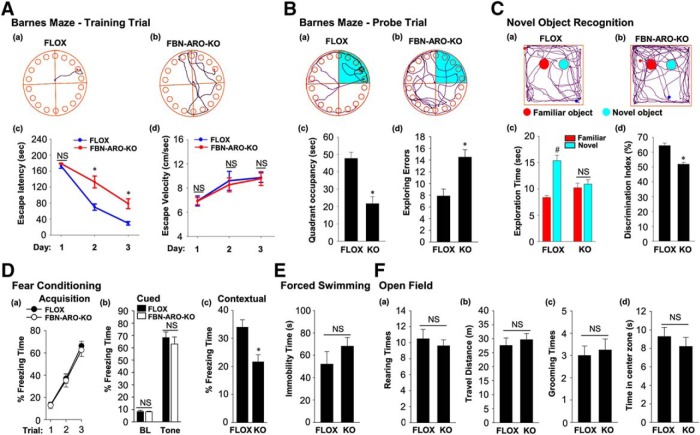
Behavioral analysis of intact female FBN-ARO-KO mice. ***Aa***, ***Ab***, Representative tracking plots of both groups on the third day of the Barnes maze training trial. Escape latency (***Ac***) and escape velocity (***Ad***) were recorded and statistically analyzed. ***Ba***, ***Bb***, Representative tracking plots of both groups on Barnes maze probe trial. Quadrant occupancy (***Bc***) and exploring errors (***Bd***) during this stage were recorded. ***C***, Representative tracking plots of both FLOX (***Ca***) and FBN-ARO-KO (***Cb***) mice on NORT choice session are shown. Exploration time (***Cc***) and discrimination index (***Cd***) were statistically analyzed. ***D***, The fear-conditioning test was conducted to determine both cued and contextual fear memory. Fear acquisition analysis during conditioning test was conducted (***Da***). There was no alteration in cued freezing rate (***Db***), but a dramatic decrease in contextual freezing rate (***Dc***) was observed in FBN-ARO-KO mice examined 24 h after fear conditioning. ***E***, The forced-swimming test was performed, with immobility time recorded as an index of depression. ***F***, The open-field test was performed to evaluate locomotor function by examining rearing times (***Fa***) and travel distance (***Fb***) and anxiety level was determined by examining grooming times (***Fc***) and time spent in center zone (***Fd***). Data are shown as means ± SEM of determinations from each group. *n* = 8. *p < 0.05 versus FLOX group. #*p* < 0.05 versus familiar object.

**Figure 5.**
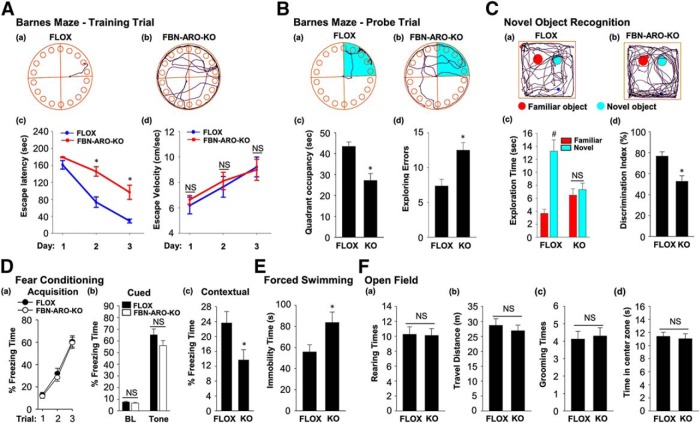
Ovx female FBN-ARO-KO mice exhibit cognitive defects but normal locomotor activity and anxiety levels. ***A***, Representative tracking plots of Barnes maze test for the indicated ovx female mice on the third day of training trial. Escape latency (***Ac***) and escape velocity (***Ad***) were recorded and statistically analyzed. ***Ba***, ***Bb***, Representative tracking plots of Barnes maze test on the probe trial day. Relative quadrant occupancy (***Bc***) and exploring errors (***Bd***) during probe trial were recorded. ***C***, Representative tracking plots of indicated groups on NORT choice day are indicated. Exploration time (***Cc***) and discrimination index (***Cd***) were statistically analyzed. ***D***, Fear acquisition analysis during conditioning test was conducted (***Da***). Cued freezing rate (***Db***) and contextual freezing rate (***Dc***) were determined 24 h after fear conditioning. ***E***, The forced-swimming test was conducted, with immobility time recorded during swimming. ***F***, The open-field test was performed with rearing times (***Fa***), travel distance (***Fb***), grooming times (***Fc***), and time in center zone (***Fd***) assayed, which showed no alterations in ovx female FBN-ARO-KO mice. Data are shown as means ± SEM of determinations from each group. *n* = 8–10. *p < 0.05 versus FLOX group; #*p* < 0.05 versus familiar object.

Next, we subjected male FBN-ARO-KO mice to the Barnes maze task, a widely used test of hippocampal-dependent spatial reference learning and memory. The results revealed that FBN-ARO-KO mice displayed a significant spatial memory defect as evidenced by an increase in escape latency to find the hidden chamber on the third day of the training trial (*p* = 0.003, two-way ANOVA), with decreased quadrant occupancy (*p* = 0.009, unpaired *t* test) and increased number of exploring errors on probe trial (*p* = 0.001, unpaired *t* test) compared with FLOX mice (Fig. 3*A*,*B*). Escape velocity was similar between the two groups, indicating that differences on escape latencies were not due to speed variations. Similar impairments of hippocampal-dependent spatial reference learning and memory were also observed in both intact female FBN-ARO-KO mice (Fig. 4*A*,*B*, *p* = 0.002 for the escape latency on the second day, *p* = 0.002 on the third day of training trial, two-way ANOVA; *p* < 0.001 for quadrant occupancy, *p* = 0.002 for exploring errors, unpaired *t* test) and ovx female FBN-ARO-KO mice (Fig. 5*A*,*B*, *p* < 0.001 for the escape latency on the second day, *p* = 0.003 on the third day of training trial, two-way ANOVA; *p* = 0.001 for quadrant occupancy and *p* = 0.003 for exploring errors, unpaired *t* test). These findings suggest that neuron-derived E2 has an important role in hippocampal-dependent spatial reference learning and memory in both male and female mice.

Using the novel object recognition test, we also found that male, intact female, and ovx female FBN-ARO-KO mice all displayed a significant impairment in hippocampal-dependent recognition memory, as evidenced by a significant decrease in preference to explore the novel object compared with the FLOX mice (Figs. 3*C*, 4*C*, 5*C*, with *p* < 0.001, *p* < 0.001, *p* = 0.004 respectively, unpaired *t* test). Exploration of long-term fear memory using the fear-conditioning test revealed that male FBN-ARO-KO mice (Fig. 3*D*), intact female FBN-ARO-KO mice (Fig. 4*D*), and ovx female FBN-ARO-KO mice (Fig. 5*D*) did not differ in fear acquisition (Fig. 3*Da*, *p* = 0.581; Fig. 4*Da*, *p* = 0.601; Fig. 5*Da*, *p* = 0.524 respectively, two-way ANOVA) and cued freezing rate (Fig. 3*Db*, *p* = 0.888; Fig. 4*Db*, *p* = 0.846; Fig. 5*Db*, *p* = 0.294 respectively for baseline, Fig. 3*Db*, *p* = 0.677; Fig. 4*Db*, *p* = 0.485; Fig. 5*Db*, *p* = 0.182 respectively for tone, unpaired *t* test) from FLOX controls, but did exhibit a significant decrease in the hippocampal-dependent contextual freezing rate (Fig. 3*Dc*, *p* = 0.038; Fig. 4*Dc*, *p* = 0.005; Fig. 5*Dc*, *p* = 0.028 respectively, unpaired *t* test). Therefore, neuron-derived E2 appears to also have an important role in the hippocampus to regulate contextual fear memory in both males and females.

We next examined depressive-like behavior in the mice using the forced-swimming test. The results revealed that there was no significant difference in immobility time in male FBN-ARO-KO mice (Fig. 3*E*, *p* = 0.593, unpaired *t* test) or intact female FBN-ARO-KO mice (Fig. 4*E*, *p* = 0.261, unpaired *t* test) compared with FLOX mice. However, immobility time in ovx female FBN-ARO-KO mice was significantly increased compared with the ovx FLOX controls (Fig. 5*E*, *p* = 0.04, unpaired *t* test), which is indicative of apparent depressive behavior. Finally, we performed the open-field test to examine locomotor activity and anxiety in FBN-ARO-KO mice. The results revealed that male FBN-ARO-KO mice (Fig. 3*F*), intact female FBN-ARO-KO mice (Fig. 4*F*), and ovx female FBN-ARO-KO mice (Fig. 5*F*) exhibited similar rearing times (Fig. 3*Fa*, *p* = 0.395; Fig. 4*Fa*, *p* = 0.721; Fig. 5*Fa*, *p* = 0.914 respectively, unpaired *t* test) and travel distance (Fig. 3*Fb*, *p* = 0.787; Fig. 4*Fb*, *p* = 0.548; Fig. 5*Fb*, *p* = 0.542 respectively, unpaired *t* test) compared with the FLOX littermates, suggesting that locomotor functions were not affected following forebrain neuronal aromatase deletion. Likewise, grooming times (Fig. 3*Fc*, *p* = 0.402; Fig. 4*Fc*, *p* = 0.798; Fig. 5*Fc*, *p* = 0.794 respectively, unpaired *t* test) and time spent in the central zone (Fig. 3*Fd*, *p* = 0.767; Fig. 4*Fd*, *p* = 0.447; Fig. 5*Fd*, *p* = 0.731 respectively, unpaired *t* test) for FBN-ARO-KO mice were also statistically indistinguishable from their FLOX controls, demonstrating that FBN-ARO-KO mice do not exhibit abnormal anxiety levels.

### Depletion of neuron-derived E2 leads to a significant decrease in dendritic spine density

**Figure 6.**
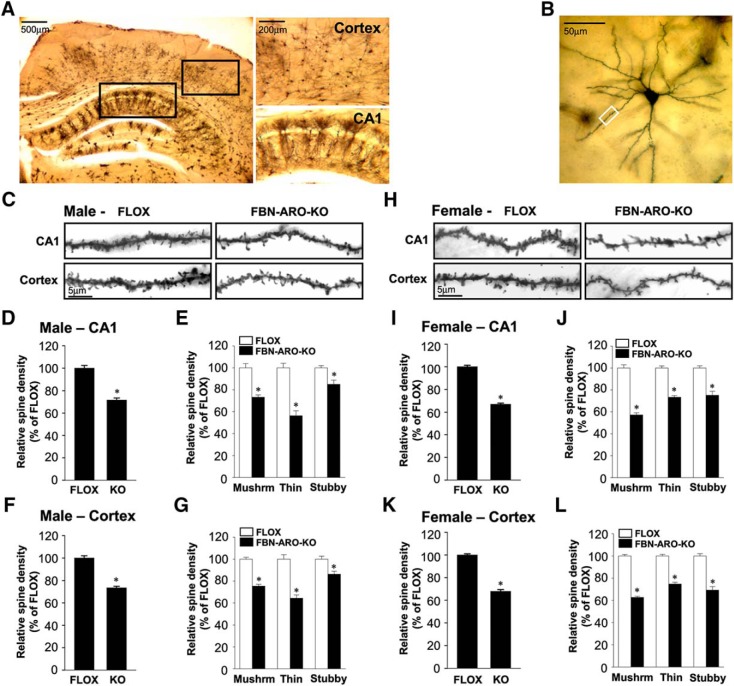
Dendritic spine density was decreased in both male and ovx female FBN-ARO-KO mice. ***A***, Representative images of coronal sections subjected to Golgi staining from cortex and hippocampal CA1 region. ***B***, Representative single neuron morphology with Golgi staining and the framed apical dendrite section for spine density analysis. ***C***, Representative images of selected dendritic segments from hippocampal CA1 region and cortex of male FLOX and FBN-ARO-KO mice. ***D***, Quantitative analysis of mean spine density in hippocampal CA1 region from male mice. ***E***, Quantitative analysis for the changes of different spines classified into mushroom, thin, and stubby based on morphology. ***F***, ***G***, Quantitative analysis of both mean spine density (***F***) and different spine morphologies (***G***) in cortex of FBN-ARO-KO mice. ***H***, Representative images of dendritic spines from hippocampal CA1 pyramidal and cortical neurons of ovx female FLOX and FBN-ARO-KO mice. ***I***, ***J***, Quantitative analysis of mean spine density (***I***) and the classified spines (***J***) in hippocampal CA1 region from ovx female mice. ***K***, ***L***, Group data of mean spine density (***K***) and classified spines (***L***) in cortex of ovx female mice. Data are shown as means ± SEM. *n* = 5. *p < 0.05 versus FLOX group. Mushrm, Mushroom.

We next examined dendritic spine formation in the mouse forebrain by using Golgi staining to visualize *in vivo* spine morphology (Fig. 6*A*). Spine density along the apical dendrite was selected for analysis (Fig. 6*B*). The results revealed that mean spine density was significantly decreased by 28.3 ± 1.8% in the hippocampal CA1 subregion (*p* < 0.001, unpaired *t* test) and by ∼26.4 ± 1.3% in the cortex (*p* < 0.001, unpaired *t* test) of male FBN-ARO-KO mice compared with FLOX mice (Fig. 6*C*,*D*,*F*). Spines were further morphologically classified into mushroom, thin, and stubby. In the hippocampal CA1 region and cortex, mushroom spines were decreased by 26.7 ± 2.0% (*p* < 0.001, unpaired *t* test) and 24.5 ± 1.6% (*p* < 0.001, unpaired *t* test), respectively, in male FBN-ARO-KO mice, whereas thin spines were decreased by 43.5 ± 4.4% (*p* < 0.001, unpaired *t* test) and 35.5 ± 2.9% (*p* < 0.001, unpaired *t* test), respectively, with stubby spines decreased by 15.0 ± 1.8% (*p* = 0.008, unpaired *t* test) and 13.7 ± 2.7% (*p* = 0.006, unpaired *t* test), respectively (Fig. 6*E*,*G*). Mean spine density in ovx female FBN-ARO-KO mice was decreased by 33.0 ± 0.9% (*p* < 0.001, unpaired *t* test) in the hippocampal CA1 region and 32.1 ± 1.5% (*p* < 0.001, unpaired *t* test) in cortex (Fig. 6*H*,*I*,*K*), with mushroom spines showing the greatest decrease: 42.7 ± 1.9% (*p* < 0.001, unpaired *t* test) in the hippocampal CA1 region (Fig. 6*J*) and 37.2 ± 0.9% (*p* < 0.001, unpaired *t* test) in the cortex (Fig. 6*L*). Density of thin and stubby spines was decreased by 26.6 ± 1.5% (*p* < 0.001, unpaired *t* test) and 24.8 ± 3.6% (*p* < 0.001, unpaired *t* test), respectively, in the hippocampal CA1 region and 25.2 ± 1.6% (*p* < 0.001, unpaired *t* test) and 30.7 ± 3.1% (*p* = 0.003, unpaired *t* test), respectively, in the cortex of ovx female FBN-ARO-KO mice. These findings indicate that neuron-derived E2 is an important regulator of spine formation in the hippocampal CA1 region and cerebral cortex in both male and female mice.

### Loss of neuron-derived E2 leads to a significant decrease in synapse number

**Figure 7.**
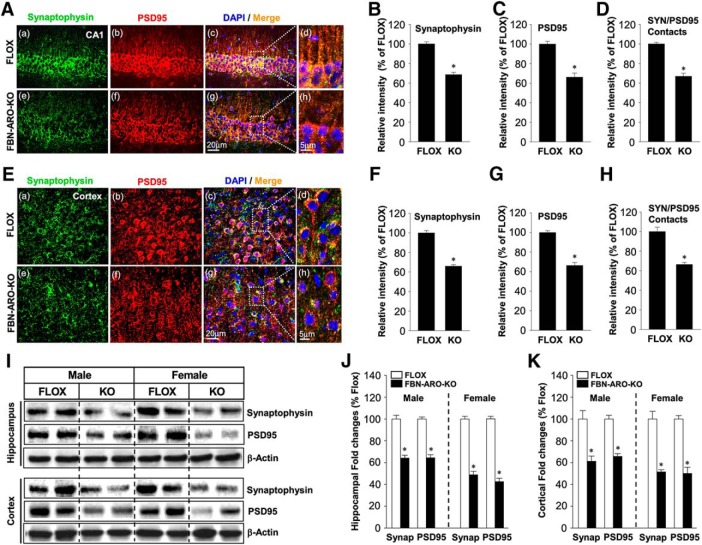
Synaptic density is significantly impaired in male FBN-ARO-KO mice. ***A***, IHC analysis for the expression and localization of both the presynaptic marker synaptophysin and the postsynaptic marker PSD95 in the male hippocampal CA1 region. ***B***–***D***, Quantitative analysis for the relative intensity of synaptophysin (SYN) (***B***), PSD-95 (***C***), and SYN/PSD-95 contacts which represents synaptic density (***D***) compared with FLOX mice. ***E***–***H***, IHC (***E***) and further quantitative analysis of synaptophysin (***F***), PSD95 (***G***), and SYN/PSD-95 contact (***H***) intensity in male cerebral cortex. ***I***, Western blot confirmation for synaptophysin and PSD95 expression in hippocampus and cortex from both male and ovx female mice. ***J***, ***K***, Quantitative analysis of Western blot analysis for both hippocampus (***J***) and cortex (***K***). Data are shown as means ± SEM. *n* = 5. *p < 0.05 versus FLOX group. SYN, Synap, Synaptophysin.

**Figure 8.**
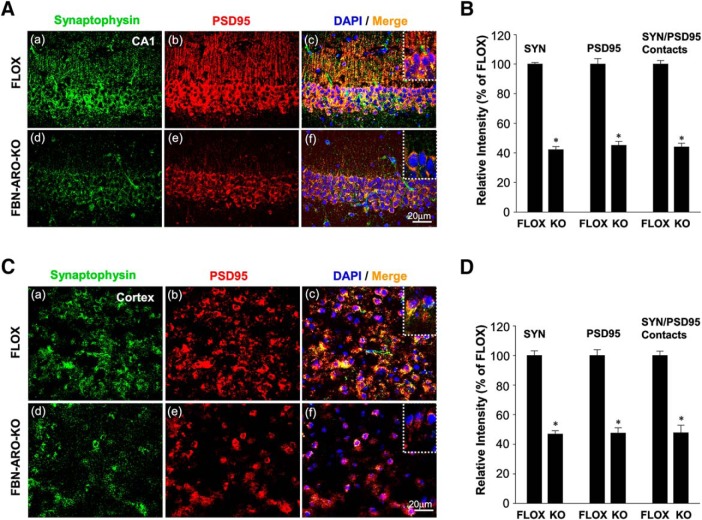
Synaptic density is decreased in ovx female FBN-ARO-KO mice. ***A***, IHC analysis for synaptophysin and PSD95 expressions, along with their localization in hippocampal CA1 subregion was performed. ***B***, Analysis of the relative intensity of synaptophysin, PSD95, and SYN/PSD95 contacts in ovx female FBN-ARO-KO versus FLOX mice. ***C***, ***D***, Changes of synaptophysin and PSD95, along with SYN/PSD95 contact intensity, in cortex of ovx female FBN-ARO-KO and FLOX mice were measured with IHC (***C***), which was further subjected to quantitative analysis in ***D***. Data are shown as means ± SEM of determinations from each group. *n* = 5. *p < 0.05 versus FLOX group. SYN, Synaptophysin.

We next investigated whether FBN-ARO-KO mice exhibit alterations in synapse number. IHC staining for the presynaptic marker synaptophysin and the postsynaptic marker PSD95 was conducted in both the hippocampal CA1 region (Fig. 7*A*) and cerebral cortex (Fig. 7*E*) to visualize presynaptic and postsynaptic specializations. Semiquantitative analysis of the IHC results showed that male FBN-ARO-KO mice exhibited a 31–33% decrease in synaptophysin (Fig. 7*B*,*F*, *p* < 0.001, unpaired *t* test) and PSD95 (Fig. 7*C*,*G*, *p* < 0.001, unpaired *t* test) in both the hippocampal CA1 and cortex. Intensity of SYN/PSD95 contacts was analyzed as an index of reactive synaptic density, which displayed a 33.1 ± 3.1% decrease in the hippocampal CA1 region (Fig. 7*D*, *p* < 0.001, unpaired *t* test) and a 33.7 ± 1.9% decrease in cortex (Fig. 7*H*, *p* < 0.001, unpaired *t* test) of male FBN-ARO-KO mice compared with FLOX controls. Studies in ovx female FBN-ARO-KO mice showed a similar robust decrease (55–58%) in synaptophysin, PSD95, and SYN/PSD95 contacts in the forebrain hippocampal CA1 region (Fig. 8*A*,*B*) and cortex (Fig. 8*C*,*D*). Western blot analysis confirmed a significant decrease in both synaptophysin and PSD95 protein levels in the hippocampus and cortex of male and female FBN-ARO-KO mice (Fig. 7*I–K*). Overall, these results indicate that neuron-derived E2 is an important regulator of forebrain synaptic density in both male and female mice.

### Functional synaptic plasticity is significantly impaired in FBN-ARO-KO mice and rescued by acute E2 treatment

**Figure 9.**
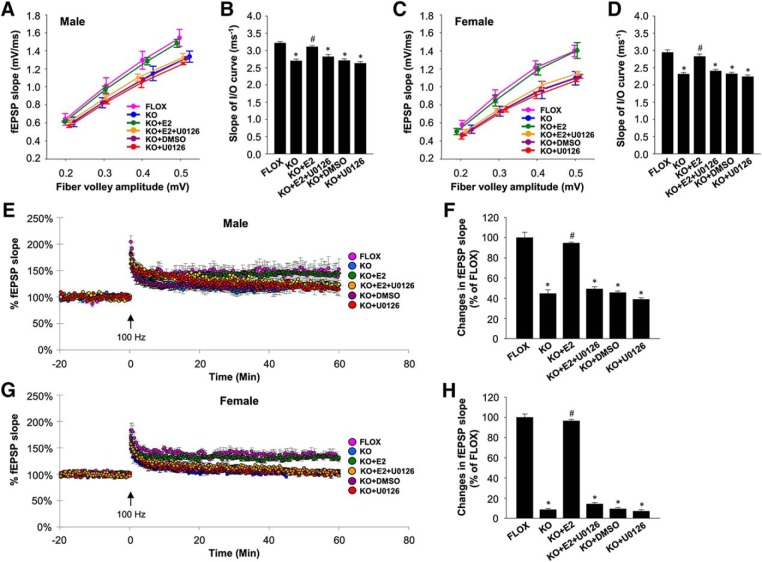
Functional synaptic plasticity is impaired in both male and ovx female FBN-ARO-KO mice and rescued by acute E2 treatment. ***A***, Examination of the alteration in excitatory synaptic transmission in male FBN-ARO-KO mice compared with the FLOX control and the rescue effect by acute E2 treatment (1 nm). In another group, U0126 (10 μm), a MEK/ERK inhibitor, was coadministered with E2 to determine the role of MEK-ERK signaling in the regulation of acute E2 benefit on synaptic transmission. ***B***, Quantification of the slopes of I/O curve obtained from linear regression of the I/O curves in ***A***. ***C***, ***D***, Analysis of excitatory synaptic transmission in ovx female mice and the rescue effect by acute E2 administration (1 nm) on FBN-ARO-KO brain slices. In another group, U0126 (10 μm) was coadministered with E2 to determine the role of MEK-ERK signaling in E2 rescue. ***C***, Corresponding changes in slope of I/O curve were quantified in ***D***. ***E***, LTP recording of Schaffer collateral synapses in above male groups by HFS (100 Hz, 1 s) stimulation, with the fEPSP slope measured for 60 min after LTP induction. ***F***, Quantitative analysis for changes in mean fEPSP slope between 50 and 60 min after LTP induction indicated in ***E***. ***G***, ***H***, LTP recording in ovx female mice (***G***) and the analysis for fEPSP slope in each group (***H***). Data are shown as means ± SEM of determinations from each group. *n* = 6 slices. *p < 0.05 versus FLOX group; *#p* < 0.05 versus KO group.

We next investigated whether loss of neuron-derived E2 results in impaired functional synaptic plasticity in the forebrain. To assess the efficacy of synaptic transmission, we examined excitatory synaptic transmission of hippocampal CA1 neurons by determining the relationship between the amplitude of presynaptic fiber volley and fEPSP slope (I/O). As shown in Figure 9*A*, the results revealed a less steep I/O relationship in male FBN-ARO-KO mice compared with the male FLOX control mice. Further analysis of the slope of the I/O curve (per millisecond) revealed that the mean slope of I/O curves in male FBN-ARO-KO mice was 2.7 ± 0.05 ms^−1^ compared with 3.2 ± 0.04 ms^−1^ in FLOX control mice (Fig. 9*B*, *p* < 0.001, two-way ANOVA), indicating that the fEPSP slope response per presynaptic action potential was decreased after neuron-derived E2 depletion. Furthermore, acute application of exogenous E2 (1 nm) on FBN-ARO-KO brain slices during electrophysiological measurement rapidly reversed the defect in synaptic transmission to a level (3.1 ± 0.03 ms^−1^) that was not different from that observed in FLOX mice (Fig. 9*B*, *p* < 0.001, two-way ANOVA). DMSO alone vehicle had no effect on synaptic transmission (Fig. 9*B*, *p* = 0.912, two-way ANOVA). Coincubation of U0126 (10 μm), an inhibitor of MEK/ERK, with E2 completely prevented the E2 effect (Fig. 9*B*, *p* = 0.009, two-way ANOVA), suggesting the rescue effects by acute E2 administration are driven via ERK signaling pathway. Note that incubation with only U0126 did not change synaptic transmission (Fig. 9*B*, *p* = 0.39, two-way ANOVA). A similar pattern of I/O relationship was found in ovx female FBN-ARO-KO mice (Fig. 9*C*), with the mean slope of the I/O curve downregulated from 2.9 ± 0.07 ms^−1^ in FLOX control to 2.3 ± 0.04 ms^−1^ (*p* < 0.001, two-way ANOVA) in the FBN-ARO-KO mice, which was fully rescued to 2.8 ± 0.08 ms^−1^ by acute 1 nm E2 administration (Fig. 9*D*, *p* = 0.002, two-way ANOVA). Coincubation of U0126 with E2 abolished the E2 rescue effect (Fig. 9*D*, *p* = 0.003, two-way ANOVA), whereas DMSO vehicle and U0126 alone had no effects on synaptic transmission (Fig. 9*D*, *p* = 0.908 and 0.322, respectively, two-way ANOVA). These results demonstrate that neuron-derived E2 is critical for maintaining efficient synaptic transmission in the forebrain of both male and female mice.

Next, we investigated LTP in the hippocampal CA1 region using HFS (100 Hz, 1 s) to induce a rapid increase in fEPSP slope, which was recorded for 60 min. As shown in Figure 9, *E* and *G*, in response to HFS, there was a robust induction of LTP in FLOX control mice. Although LTP induction still occurred in both male FBN-ARO-KO mice (Fig. 9*E*) and ovx female FBN-ARO-KO mice (Fig. 9*G*), the amplitude of LTP was significantly impaired. Quantitative analysis of mean fEPSP slope between 50 and 60 min of LTP recording showed a 57 ± 2.3% decrease in male FBN-ARO-KO mice (Fig. 9*F*, *p* < 0.001, two-way ANOVA) and a 91.4 ± 1.9% decrease in ovx female FBN-ARO-KO mice (Fig. 9*H*, *p* < 0.001, two-way ANOVA) compared with their FLOX littermate controls. We next investigated whether acute *in vitro* E2 treatment of the hippocampal slices could rescue the LTP impairment in the FBN-ARO-KO mice. The results showed that acute E2 application (1 nm) was able to rapidly rescue LTP amplitude in FBN-ARO-KO mice. In males, the fEPSP slope in FBN-ARO-KO mice was increased to 94.6 ± 0.95% of FLOX level by E2 replacement (Fig. 9*F*, *p* < 0.001, two-way ANOVA), whereas the level in ovx female FBN-ARO-KO mice was reinstated to 96.54 ± 1.4% of their FLOX control (Fig. 9*H*, *p* < 0.001, two-way ANOVA). DMSO alone vehicle had no effect on LTP amplitude (*p* = 0.823 for males, *p* = 0.671 for females, two-way ANOVA). Coadministration of U0126 with E2 blocked the ability of E2 to rescue LTP amplitude in both males (Fig. 9*F*, *p* < 0.001, two-way ANOVA) and females (Fig. 9*H*, *p* < 0.001, two-way ANOVA). U0126 treatment alone had no significant effect on LTP in either male FBN-ARO-KO mice (Fig. 9*F*, *p* = 0.125, two-way ANOVA) or female FBN-ARO-KO mice (Fig. 9*H*, *p* = 0.508, two-way ANOVA). These results demonstrate that forebrain neuronal E2 is necessary to maintain full expression of LTP in the hippocampus of both male and female mice. It also provides direct evidence that this beneficial E2 effect can be achieved through rapid nongenomic modulation involving the MEK-ERK signaling pathway.

### Rapid kinase and neurotrophic signaling is significantly attenuated in FBN-ARO-KO mice

**Figure 10.**
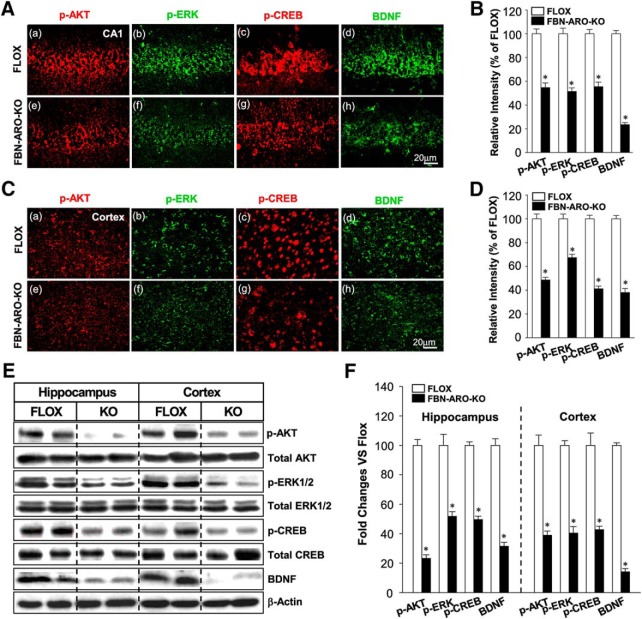
Male FBN-ARO-KO mice exhibit significant defects in forebrain AKT-ERK and CREB-BDNF signaling. ***A***, ***B***, IHC staining (***A***) and the quantification (***B***) of p-AKT, p-ERK, p-CREB and BDNF levels in hippocampal CA1 region. ***C***, ***D***, The same parameters in cerebral cortex were examined with confocal analysis (***C***) and further quantified in ***D***. ***E***, ***F***, Western blot (***E***) was subsequently performed to confirm the variations shown above and further quantified in (***F***), which shows the same consistent pattern as observed with IHC staining. Data are shown as means ± SEM of determinations from each group. *n* = 5. *p < 0.05 versus FLOX group.

**Figure 11.**
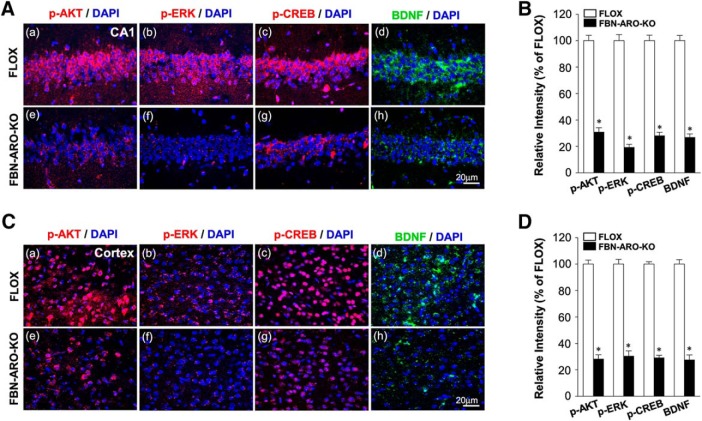
Ovx female FBN-ARO-KO mice exhibit significant defects in forebrain AKT-ERK and CREB-BDNF signaling. ***A***, Representative confocal images of p-AKT, p-ERK, p-CREB, and BDNF in hippocampal CA1 regions from the FLOX and FBN-ARO-KO groups in ovx female mice. ***B***, Confocal analysis in ***A*** was further quantified in a diagram form with data expressed as percentage changes of each group versus FLOX group. ***C***, ***D***, Cerebral cortex was subjected to IHC analysis (***C***) to detect the changes of p-AKT, p-ERK, p-CREB, and BDNF in FBN-ARO-KO mice. Further quantitative analysis was conducted, with percentage changes versus FLOX mice shown in ***D***. Data are shown as means ± SEM of determinations from each group. *n* = 5. *p < 0.05 versus FLOX group.

To further confirm and extend the *in vitro* results, we next examined rapid AKT-ERK kinase and CREB-BDNF neurotrophic signaling in the hippocampal CA1 and cortex region of FBN-ARO-KO mice (Fig. 10*A–F*). IHC results showed that the phosphorylation of AKT, ERK, and CREB in the hippocampal CA1 region of male FBN-ARO-KO mice was decreased by ∼44.5–48.6%, whereas the BDNF levels were decreased by 76.5 ± 1.7% compared with the FLOX control (Fig. 10*A*,*B* with *p* < 0.001, *p* = 0.013, *p* = 0.007, *p* < 0.001, respectively, unpaired *t* test). A similar pattern was observed in cortex with a 32.5–58.7% decrease in the phosphorylation of AKT, ERK, CREB, and a 61.9 ± 3.4% reduction in BDNF levels (Fig. 10*C*,*D*, *p* < 0.001, *p* = 0.022, *p* = 0.031,and *p* = 0.007, respectively, unpaired *t* test). Western blot analysis confirmed a similar robust decrease in pAKT, pERK, pCREB, and BDNF levels in both the hippocampus and cerebral cortex of male FBN-ARO-KO mice (Fig. 10*E*,*F*). Likewise, studies in ovx female FBN-ARO-KO mice revealed a similar 69.1–80.7% decrease in pAKT, pERK, pCREB, and BDNF levels in the hippocampus (Fig. 11*A*,*B*, *p* < 0.001, *p* = 0.023, *p* = 0.002, *p* = 0.005, respectively, unpaired *t* test) and a 69.6–72.5% decrease in the cortex compared with their FLOX controls (Fig. 11*C*,*D*, *p* = 0.004, *p* = 0.026, *p* = 0.002, and *p* < 0.001 respectively, unpaired *t* test).

### *In vivo* exogenous E2 replacement rescues the molecular and functional deficits in FBN-ARO-KO mice

**Figure 12.**
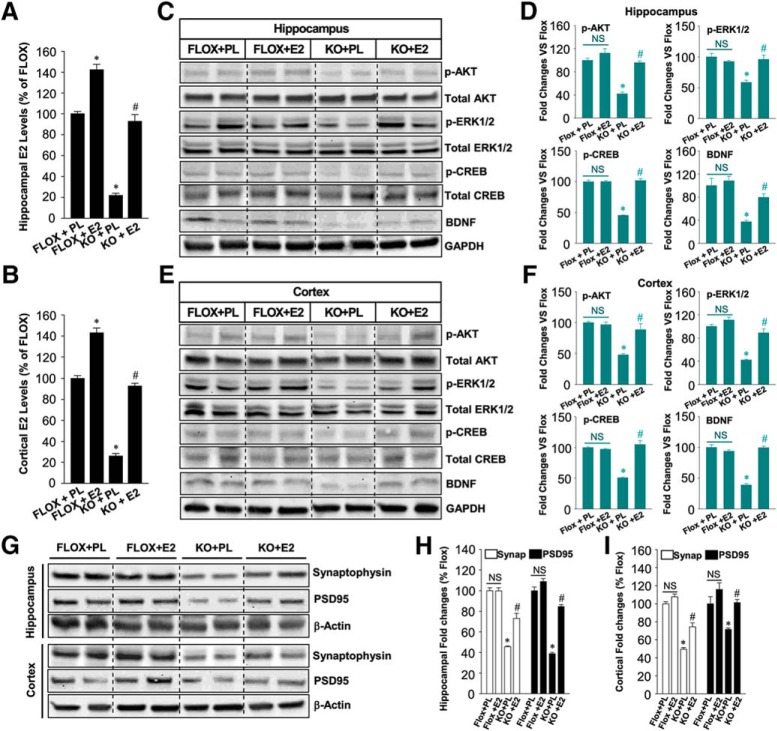
Exogenous *in vivo* E2 replacement “rescues” AKT-ERK-CREB-BDNF signaling and synaptic density in ovx female FBN-ARO-KO mice. ***A***, ***B***, E2 levels in hippocampus (***A***) and cortex (***B***) of FLOX+PL, FLOX+E2, KO+PL, and KO+E2 mice were measured with E2 ELISA after E2 replacement. Data were quantified as percentage changes compared with the FLOX+PL group. ***C***–***F***, E2 replacement on the activation of neurotrophic signaling pathway in both hippocampus (***C***) and cortex (***E***) was determined through Western blot, which was probed with phospho- and total AKT, ERK1/2, CREB, and BDNF antibodies. Western blot in ***C*** and ***E*** was quantified in ***D*** and ***F***, respectively, with data expressed as percentage changes versus FLOX+PL group. ***G***–***I***, Hippocampal and cortical lysates from the indicated groups were subjected to Western blot for synaptophysin and PSD95 expression to examine the effect of E2 replacement on synaptic restoration in FBN-ARO-KO mice (***G***). The result was further quantified as percentage changes of each group versus FLOX+PL group (***H***, ***I***). Data are shown as means ± SEM. *n* = 4–5. *p < 0.05 versus FLOX+PL group; #*p* < 0.05 versus KO+PL group. PL, Placebo; Synap, synaptophysin.

**Figure 13.**
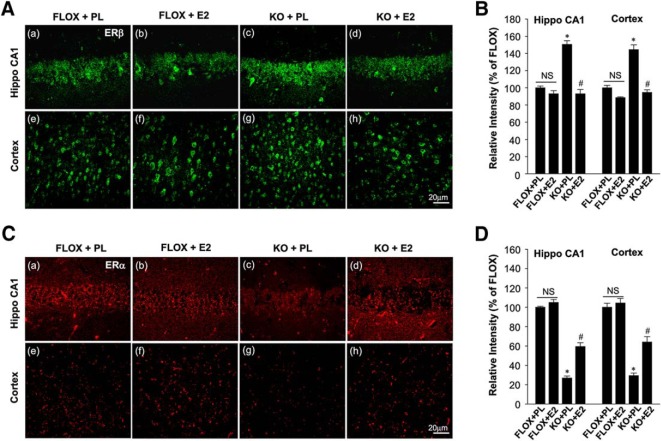
Alterations of ER-α and ER-β levels in the forebrain of ovx female FBN-ARO-KO mice and effect of exogenous *in vivo* E2 replacement. ***A***, Representative confocal images of ER-β in both hippocampal CA1 and cortex of the indicated groups subjected to IHC analysis. ***B***, Confocal analysis in ***A*** was further quantified as relative changes of each group versus FLOX+PL group. ***C***, ***D***, Representative confocal images of ER-α in hippocampal CA1 region and cerebral cortex from the indicated groups (***C***). Confocal images were further quantified in (***D***). Data are shown as means ± SEM of determinations from each group. *n* = 4–5. *p < 0.05 versus FLOX+PL group; #*p* < 0.05 versus FBN-ARO-KO + PL group. Hippo, Hippocampus; PL, placebo.

We next investigated whether *in vivo* exogenous E2 replacement could rescue the molecular and functional deficits in ovx FBN-ARO-KO mice. As shown in Figure 12, *A* and *B*, E2 replacement in ovx FBN-ARO-KO mice was able to reinstate hippocampal and cortical E2 levels to 93% of the FLOX control (*p* = 0.005, *p* < 0.001, respectively, two-way ANOVA). Western blot results revealed that the phosphorylation of AKT, ERK, and CREB in FBN-ARO-KO mice were restored to 90–95% in the hippocampus (*p* = 0.007, *p* < 0.001, *p* = 0.002, respectively, two-way ANOVA) and cortex (*p* = 0.007, *p* = 0.003, *p* = 0.007, respectively, two-way ANOVA) by E2 replacement, whereas BDNF levels were increased to 80–90% of FLOX control levels (Fig. 12*C–F*, *p* = 0.012 and *p* < 0.001, respectively, two-way ANOVA), indicating an almost complete rescue of these signaling pathways. Western blot analysis for synaptogenesis showed that the expression of both synaptophysin and PSD95 in the forebrain was also robustly rescued by E2 replacement in ovx FBN-ARO-KO mice (Fig. 12*G–I*). This appeared to be a true “rescue“ and not just an enhancement mediated by E2 in normal forebrain because exogenous E2 replacement was able to rescue the BDNF levels and phosphorylation of AKT, ERK, and CREB in FBN-ARO-KO mice, but had no significant effect in FLOX mice. Based on these results, we hypothesized that the FBN-ARO-KO mouse forebrain may be more sensitive to E2 replacement. In support of this hypothesis, we found that ER-β, which is critical for the modulation of synaptic plasticity and memory, was upregulated by 40–50% in the hippocampal CA1 region and cerebral cortex of FBN-ARO-KO mice (Fig. 13*A*,*B*, *p* < 0.001 and *p* = 0.008, respectively, two-way ANOVA), whereas, in contrast, ER-α was markedly downregulated compared with FLOX controls (Fig. 13*C*,*D*, *p* = 0.004 and *p* < 0.001 respectively, two-way ANOVA). Exogenous E2 replacement in FBN-ARO-KO mice was able to significantly reverse these changes in both ER-α and ER-β.

**Figure 14.**
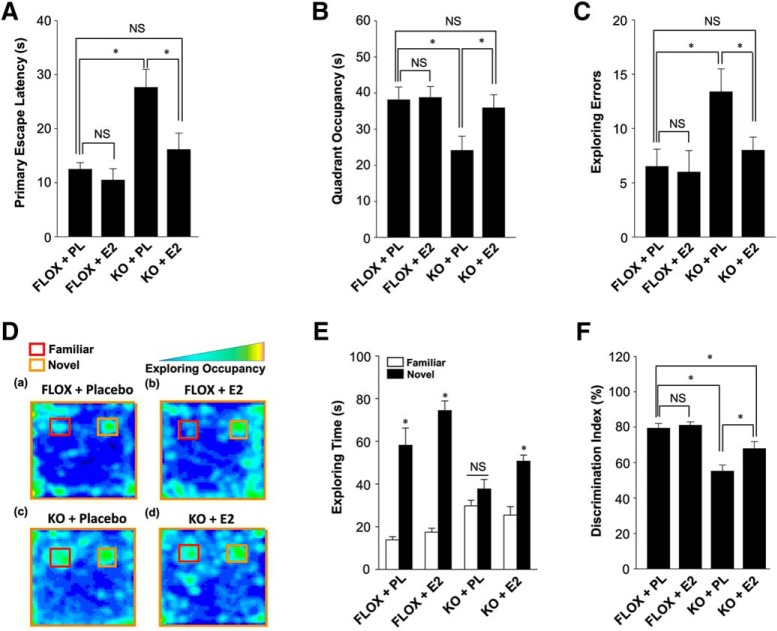
E2 replacement “rescues” hippocampal-dependent cognitive function in ovx female FBN-ARO-KO mice. ***A***, Primary escape latency for the indicated mice during Barnes maze probe trial. ***B***, Time spent in target quadrant during probe trial. ***C***, Analysis for exploring errors of the indicated mice during the Barnes maze probe trial. ***D***, Representative occupancy plots of the indicated groups during the choice session of the novel object recognition test. ***E***, Exploring time for familiar and novel objects during the choice session was recorded, and further quantified in a diagram form. ***F***, Result in ***E*** was further converted to discrimination index, a parameter of exploring rate on novel object. Values are means ± SEM of determinations from each group. *n* = 8. *p < 0.05. PL, Placebo.

Next, we performed the Barnes maze test and novel object recognition test to determine whether reinstating forebrain E2 levels via E2 replacement could rescue functional outcome in FBN-ARO-KO mice. As shown in Figure 14, E2 replacement was able to rescue the spatial learning and memory defects as evidenced by E2-treated ovx FBN-ARO-KO mice exhibiting a significantly decreased primary escape latency (Fig. 14*A*), spending more time in the target quadrant exploring the removed hole (Fig. 14*B*), and having decreased exploring errors during the probe trial stage (Fig. 14*C*) compared with the placebo-treated ovx FBN-ARO-KO mice. Using the novel object recognition test, we found that E2 could rescue recognition memory because occupancy plots in Figure 14*D* showed that E2 replacement of ovx FBN-ARO-KO resulted in significantly improved preference to the novel object. Quantitative analysis confirmed that E2 treated FBN-ARO-KO mice spent more time exploring the novel object than FBN-ARO-KO mice treated with placebo (Fig. 14*E*,*F*). Collectively, our data demonstrate that restoration of forebrain E2 levels by exogenous E2 replacement is capable of rescuing both the molecular and functional deficits in ovx FBN-ARO-KO mice.

## Discussion

The discovery that aromatase is constitutively expressed in forebrain neurons of both male and female in many species and can produce significant levels of E2 via rapid regulation of phosphorylation indicates that neuron-derived E2 may have important roles in synaptic transmission and brain function. By creating FBN-ARO-KO mice, the current study demonstrated the following: (1) loss of neuron-derived E2 in the forebrain induced significant impairment in hippocampal-dependent cognitive functions in both male and female mice; (2) neuron-derived E2 deficiency led to a significant decrease in dendritic spine density and spine synapses that was associated with attenuated rapid kinase AKT-ERK and CREB-BDNF neurotrophic signaling in the forebrain; (3) reinstating forebrain E2 levels through *in vivo* exogenous E2 replacement fully rescued these molecular and functional defects in FBN-ARO-KO mice; and (4) *in vitro* studies revealed that LTP amplitude was impaired in FBN-ARO-KO mice and could be fully rescued by acute E2 treatment, an effect dependent upon rapid ERK signaling.

When using KO mouse models, one must be concerned with the possibility that the phenotype observed could be due to altered or abnormal brain development. However, this seems unlikely in our study because the Cre recombinase in our FBN-ARO-KO mouse model is expressed specifically in forebrain neurons after postnatal day 18–23 (under the control of the CaMKIIα gene promoter) and previous studies have shown that forebrain neurons are already well differentiated by postnatal day 7, with fully established neuronal connections (Stanfield and Cowan, 1979; Pokorný and Yamamoto, 1981a,b). Furthermore, many studies, including the brain structure data provided here, have shown that development of the brain is normal in CaMKIIα-Cre forebrain KO models (Burgin et al., 1990; Tsien et al., 1996; Tsien, 1998; Kusakari et al., 2015). Finally, our study showed that acute treatment of FBN-ARO-KO slices with E2 significantly improved the deficit in synaptic transmission efficiency and completely reversed the deficit in LTP within minutes. Therefore, the plasticity and behavioral deficits in FBN-ARO-KO mice do not simply reflect postnatal developmental consequences of the neuronal aromatase KO, but rather reflect an acute requirement for neuronal aromatase and neuronal-derived E2 in ongoing synaptic function in the hippocampus.

An important observation of our study is that male and female FBN-ARO-KO mice displayed significant defects in spatial learning and memory, recognition memory, and contextual fear memory. These differences were not due to differences in locomotor function because we found that both male and female FBN-ARO-KO mice had normal locomotor function and anxiety levels. Our findings suggest that neuron-derived E2 plays a critical role in hippocampal-dependent cognitive functions in both male and female mice. A similar role may exist for neuron-derived E2 in humans because widespread expression of aromatase has been reported in neurons in the human hippocampus and cortex (Yague et al., 2010). Furthermore, clinical studies have demonstrated that postmenopausal women who are subjected to systemic aromatase inhibitor treatment for advanced breast cancer tend to suffer from a variety of memory defects (Bender et al., 2007; Phillips et al., 2011; Underwood et al., 2018), including impaired hippocampal-dependent memory and decreased hippocampal activity during encoding (Bayer et al., 2015), although there are dissenting studies (Hurria et al., 2014; Le Rhun et al., 2015). Aromatase inhibitor administration has also been reported to lead to memory defects in mice and songbirds (Bailey et al., 2017; Zameer and Vohora, 2017).

Our study also revealed a significant decrease in dendritic spine and synaptic density in the forebrains of both male and female FBN-ARO-KO mice. Interestingly, a previous study (Zhou et al., 2010) reported that letrozole treatment decreased hippocampal spines and synapse density in female but not in male mice, suggesting that neuron-derived E2 effects on spines and synaptic density were gender specific. The reason for this discrepancy between our findings and theirs is unclear. However, another group that used a twofold higher dose of letrozole did find a significant loss of dendritic spines and synapses in the hippocampus of male mice (Zhao et al., 2018).

An intriguing question is whether the loss of spines and synapses observed in FBN-ARO-KO mice causes the LTP impairment observed in the FBN-ARO-KO mice. We believe that this is not the case because our *in vitro* studies demonstrated that E2 could rescue LTP amplitude within minutes, an effect too rapid for the induction of spine synapses to have occurred. A similar conclusion was reached by Vierk et al. (2012) in their studies using the aromatase inhibitor letrozole in female mice: they found that LTP impairment preceded hippocampal spine and synapse loss in letrozole-treated mice. Based on these findings, we propose that the spine and synapse loss observed in FBN-ARO-KO mice in our study may result from impaired LTP. In support of this hypothesis, several studies have demonstrated that LTP can induce the formation of spines and synapses (Toni et al., 1999; Yuste and Bonhoeffer, 2001).

Our findings also provide insight into the potential signaling mechanisms that underlie neuron-derived E2 effects on LTP, synaptic plasticity, and memory. Our discovery that the activation of both AKT and ERK is significantly attenuated in the forebrain of FBN-ARO-KO mice suggests that neuron-derived E2, most likely acting through an extranuclear signaling mechanism, can regulate these rapid kinase signaling pathways. Both AKT and ERK signaling has been implicated to help mediate LTP, synaptic plasticity, and memory (Sweatt, 2001; Giovannini, 2006; Levenga et al., 2017). Furthermore, E2 is well known to enhance both AKT and ERK signaling in the hippocampus and cortex through rapid extranuclear ER-mediated signaling (Raz et al., 2008). In addition, E2-induced LTP induction can be blocked by inhibiting ERK and PI3K-AKT signaling in male rat hippocampal slices (Hasegawa et al., 2015) and our own study showed that the ability of acute E2 to rescue LTP in hippocampal slices from FBN-ARO-KO mice could be abolished by inhibiting ERK signaling. Based on these findings, we propose that rapid extranuclear neuronal E2 signaling regulates ERK and AKT activation in the forebrain, which may contribute significantly to its effects upon LTP, synaptic plasticity, and memory.

Our study also revealed a significant decrease of pCREB and BDNF expression in the FBN-ARO-KO forebrain. CREB is a major transcription factor known to play an important role in learning and memory (Lonze and Ginty, 2002). BDNF is a neurotrophin that plays an important role in the regulation of synaptic transmission, LTP, and memory via effects mediated by TrkB receptors that are coupled to activation of the ERK and PI3K-AKT pathways (Korte et al., 1996; Ying et al., 2002; Lu et al., 2008; Leal et al., 2014). In neurons, E2 has been reported to induce the phosphorylation of CREB via the ERK pathway (Szego et al., 2006) and phosphorylated CREB can act in the nucleus to enhance the expression of BDNF (Tao et al., 1998). Indeed, previous work has demonstrated that E2 can enhance BDNF in the rodent forebrain (Luine and Frankfurt, 2013). Interestingly, BDNF-heterozygous KO mice have impaired LTP, memory, and synaptic plasticity (Korte et al., 1995; Liu et al., 2004), which mirrors our findings in FBN-ARO-KO mice. Collectively, our findings highlight the importance of neuron-derived E2 in maintaining BDNF levels, as well as activation of AKT, ERK, and CREB in the forebrains of male and female mice.

Finally, in addition to demonstrating the ability of acute E2 treatment to rescue LTP amplitude *in vitro*, we also demonstrated that *in vivo* E2 replacement that restores forebrain E2 levels in the FBN-ARO-KO mice could rescue the synaptic and behavioral deficits in ovx female FBN-ARO-KO mice. In addition, *in vivo* E2 rescue also reinstated pAKT, pERK, pCREB, and BDNF levels, as well as synaptophysin and PSD95 expression, in the forebrain of FBN-ARO-KO mice. Furthermore, *in vivo* E2 replacement also rescued the spatial and recognition memory defects in FBN-ARO-KO mice. These E2 effects appears to be true “rescue” effects and not just an enhancement mediated by E2 in normal forebrain because exogenous E2 replacement had no significant effect in FLOX mice. Interestingly, we found that ER-β was upregulated in the FBN-ARO-KO mouse forebrain, whereas ER-α was downregulated. ER-β has been shown to be critical for E2 effects upon PSD95, LTP, synaptic plasticity, and memory in mice (Liu et al., 2008). Therefore, upregulation of ER-β could make the FBN-ARO-KO mouse forebrain more sensitive to E2. Additional studies are needed to further explore this possibility.

In conclusion, our study demonstrates that forebrain-neuron-derived E2 regulates synaptic plasticity and hippocampal-dependent cognitive function in both male and female mice. Neuron-derived E2 was also demonstrated to be essential for the normal expression of LTP in both male and female mouse hippocampal slices *in vitro* and acute E2 treatment was able to rapidly and fully rescue the LTP defect in an ERK-dependent manner. Neuron-derived E2 was also demonstrated to be important for maintaining BDNF levels, as well as activation of ERK, AKT, and CREB in the forebrains of male and female mice. Collectively, our results support an important role for neuron-derived E2 as a novel “neuromodulator” in both the male and female forebrain to regulate LTP, synaptic plasticity, and cognitive function.
